# Tomato Leaf Color Diversity as a Functional Trait: Molecular Mechanisms, Physiological Significance, and Environmental Responses

**DOI:** 10.3390/ijms27146151

**Published:** 2026-07-09

**Authors:** Rahmatullah Jan, Shahzad Iqbal, Sajad Ali, Kyung-Min Kim

**Affiliations:** 1 Coastal Agriculture Research Institute, Kyungpook National University, Daegu 41566, Republic of Korea; rahmat2021@knu.ac.kr; 2 Department of Semiconductor Engineering, Gachon University, Seongnamdaero, Sujeong-gu, Seongnam-si 13120, Republic of Korea; shahzad55@gachon.ac.kr; 3 Department of Biological Sciences, College of Science, King Faisal University, Al-Ahsa 31982, Saudi Arabia; 4 Department of Applied Biosciences, Graduate School, Kyungpook National University, Daegu 41566, Republic of Korea

**Keywords:** tomato, leaf coloration, pigment metabolism, chloroplast biogenesis, multi-omics

## Abstract

Leaf color in tomato (*Solanum lycopersicum* L.) is a complex and highly informative trait that reflects pigment metabolism, chloroplast development, genetic regulation, hormonal signaling, and environmental influences. This review synthesizes current knowledge on the biological basis and diversity of tomato leaf coloration, with a particular focus on the roles of chlorophylls, carotenoids, anthocyanins, and flavonoids in generating distinct visual phenotypes. It further discusses the molecular and physiological mechanisms associated with key leaf color types, including dark green, pale green, chlorotic, purple, albino, and variegated leaves, and describes how these phenotypes develop through coordinated regulation of pigment biosynthesis, chloroplast biogenesis, and stress-responsive pathways. The review also summarizes the effects of environmental factors such as light, temperature, water availability, nutrient status, salinity, heavy metals, and biotic stress on leaf pigmentation through changes in photosynthetic efficiency and oxidative balance. In addition, hormonal regulation of leaf color is discussed with emphasis on the roles of abscisic acid (ABA), ethylene (ET), cytokinins (CKs), auxins, jasmonic acids (JA), and salicylic acid (SA) in regulating chlorophyll retention and senescence-associated color transitions. Importantly, leaf coloration functions not only as a morphological trait but also as a sensitive biomarker of plant physiological status, enabling early detection of nutrient deficiencies, abiotic stress, and disease. Recent advances in multi-omics approaches, imaging technologies, and machine learning have significantly improved the understanding of the regulatory networks controlling leaf pigmentation and their relationship with crop performance. However, important gaps remain in integrating molecular mechanisms with whole-plant and field-level responses. Future progress will depend on combining systems biology, high-throughput phenotyping, and predictive modeling to translate leaf color studies into practical applications for improving tomato productivity, stress resilience, and climate adaptation.

## 1. Introduction

### 1.1. Importance of Leaf Color in Plant Biology

Leaf color is one of the most visible and informative phenotypic traits in plants, reflecting the integrated effects of developmental, physiological, genetic, and environmental processes that influence plant growth and productivity [1]. Beyond its aesthetic significance, leaf coloration serves as a dynamic physiological indicator of photosynthetic performance, pigment metabolism, nutrient status, and adaptive responses to environmental stress [2]. The green coloration that predominates in most plant leaves primarily results from chlorophyll accumulation within chloroplasts. However, phenotypic variation resulting in yellow, purple, red, white, bronze, or variegated forms arises from quantitative and qualitative alterations in chlorophylls, carotenoids, anthocyanins, flavonoids, and other secondary metabolites [3,4]. Recent advances in plant omics technologies, including transcriptomics, metabolomics, and chloroplast proteomics, have significantly enhanced the understanding of molecular mechanisms controlling leaf pigmentation and their interactions with environmental signaling pathways [5]. Consequently, leaf color has emerged not only as a morphological descriptor but also as an integrative biological trait that reflects cellular metabolism and whole-plant performance [6].

### 1.2. Leaf Coloration as an Indicator of Physiological Status

Leaf coloration is increasingly recognized as a sensitive indicator of plant physiological status and environmental responsiveness. Changes in leaf color frequently precede visible symptoms of growth inhibition and therefore provide valuable insights into internal metabolic adjustments [2,7]. Alterations in chlorophyll concentration often reflect changes in photosynthetic capacity, nitrogen availability, chloroplast development, or oxidative balance [8]. In contrast, the accumulation of anthocyanins and carotenoids commonly serves as a protective mechanism against excessive light, temperature extremes, nutrient deficiencies, heavy metal exposure, drought, salinity, and other abiotic stresses [9]. At the molecular level, these color transitions are regulated through coordinated expression of pigment biosynthetic genes, transcription factors, hormone-mediated signaling, reactive oxygen species (ROS) homeostasis, and retrograde communication between chloroplasts and the nucleus [10,11]. Recent studies further demonstrate that leaf spectral properties and color indices can function as non-destructive biomarkers for assessing plant health, enabling early diagnosis of physiological disorders and supporting technology-assisted agriculture approaches [12].

### 1.3. Economic and Agronomic Relevance in Tomato

Tomato (*Solanum lycopersicum* L.) is among the world’s most economically important horticultural crops and serves as a model system for studying pigment metabolism, stress physiology, and developmental regulation [13,14]. In tomato production systems, leaf color directly influences canopy photosynthetic efficiency, biomass accumulation, fruit yield, and overall crop quality [15]. Variations in tomato leaf coloration may result from genetic mutations, nutrient imbalance, pathogen infection, environmental stress, chloroplast dysfunction, or developmental transitions, each with significant agronomic implications [16]. Dark green leaves are often associated with enhanced chlorophyll content and improved photosynthetic competence, whereas chlorosis, purple pigmentation, bronzing, or variegation frequently indicate physiological disturbances that compromise productivity [17]. Moreover, modern breeding programs increasingly utilize leaf color traits as phenotypic markers for selecting stress-resilient cultivars, improving nutrient-use efficiency, and developing varieties better adapted to changing climatic conditions [18]. Integration of high-throughput phenotyping and molecular breeding has further strengthened the importance of leaf color as a measurable trait in tomato improvement strategies [19].

### 1.4. Scope and Objectives of the Review

Given the expanding interest in plant phenomics and agriculture, a comprehensive synthesis of tomato leaf color diversity and its mechanisms is timely and necessary. Literature for this review was retrieved from major scientific databases including Web of Science, Scopus, and Google Scholar using relevant keywords such as tomato leaf color, chlorosis, chlorophyll metabolism, chloroplast development, and plant pigmentation. The search primarily covered studies published in the last two decades, with inclusion of both tomato-specific studies and cross-species evidence when mechanistically relevant. This review aims to systematically examine reported tomato leaf color phenotypes and summarize their physiological, biochemical, genetic, and molecular determinants. Particular emphasis is given to pigment biosynthesis and degradation, chloroplast development, hormonal regulation, environmental interactions, and stress-responsive signaling pathways that collectively determine leaf coloration. Additionally, the review discusses emerging analytical approaches, including imaging technologies, omics platforms, and molecular breeding tools, that are advancing the understanding of leaf color regulation. By integrating recent findings from multiple disciplines, this review seeks to establish leaf coloration as a functional indicator of plant performance and to identify future research directions for improving tomato productivity and resilience.

## 2. Biological Basis of Tomato Leaf Coloration

Leaf coloration in vegetable crops, including tomato, is a complex trait regulated by the coordinated development of leaf tissues, chloroplast biogenesis, pigment biosynthesis, and their dynamic interactions. It reflects the integration of structural and biochemical processes at the cellular and subcellular levels [16]. As a model horticultural species, the tomato has contributed significantly to understanding the mechanisms through which leaf pigmentation integrates developmental regulation with metabolic and environmental signals [20]. In tomato, naturally occurring and induced leaf-color mutants have demonstrated that defects in chloroplast development and chlorophyll metabolism are the predominant causes of leaf color variation [16]. In contrast, anthocyanin accumulation usually occurs only in specific genetic backgrounds or under environmental stress [21]. The visible spectrum of leaf color results from the relative abundance, spatial distribution, and physiological status of multiple pigment classes, primarily chlorophylls, carotenoids, anthocyanins, and flavonoids, which collectively determine green and non-green phenotypes observed under diverse genetic and environmental conditions [22,23].

### 2.1. Development and Structure of Tomato Leaves

Tomato leaves develop through a highly regulated process initiated at the shoot apical meristem, where leaf primordia are formed and subsequently differentiate into complex, pinnately compound structures [24]. Leaf morphogenesis involves coordinated cell division, cell expansion, and vascular patterning, resulting in a mature lamina optimized for efficient light capture and gas exchange [25]. Unlike the simple leaves of Arabidopsis, tomato develops compound leaves, providing a useful model for investigating the coordination between leaf morphogenesis and chloroplast differentiation during leaf development [26]. The structural organization of the mesophyll, particularly the differentiation of palisade and spongy tissues, plays a central role in light absorption and internal light scattering, influencing the perceived leaf color [27]. Genetic regulators such as KNOTTED1-LIKE homeobox (KNOX) and TEOSINTE BRANCHED1/CYCLOIDEA/PCF (TCP) transcription factors have been implicated in determining leaf architecture, which indirectly affects pigment distribution and intensity [28]. Environmental factors, including light quality and nutrient availability, further modulate leaf expansion and tissue differentiation, ultimately contributing to variation in leaf coloration patterns [29,30].

### 2.2. Chloroplast Development and Differentiation

Chloroplast development is a major determinant of leaf greening and coloration in tomato. It involves a highly coordinated transition from undifferentiated proplastids in young tissues to fully functional chloroplasts in mature leaves. This process involves the activation of plastid genome expression, assembly of photosynthetic complexes, and synthesis of chlorophyll-protein complexes [31]. Current evidence suggests that chloroplast biogenesis is controlled through complex interactions between nuclear-encoded transcription factors and plastid-to-nucleus retrograde signaling pathways, which collectively coordinate organelle development and functionality [10]. In tomato, mutations affecting chloroplast development commonly produce chlorotic, albino, or white-viridescent phenotypes [32], whereas such mutations in Arabidopsis often result in green-white variegation, indicating that conserved chloroplast biogenesis pathways can generate visible phenotypes among species [33]. Disruptions in chloroplast development, caused by genetic mutations or environmental stresses, often result in visible pigmentation phenotypes such as chlorosis or variegation [34]. Moreover, chloroplast ultrastructure, including thylakoid membrane organization and grana stacking, directly influences light absorption efficiency and pigment stability, contributing to variation in leaf color intensity and uniformity [35].

### 2.3. Major Pigments Determining Leaf Color

Leaf coloration is primarily regulated by the composition and relative abundance of major pigment classes, each contributing distinct optical and physiological functions [36]. Chlorophylls are the dominant pigments responsible for green coloration and are essential for light capture in photosynthesis. Their biosynthesis occurs through the tetrapyrrole pathway, involving sequential enzymatic reactions from glutamyl-tRNA reduction to chlorophyll formation, with key steps mediated by magnesium chelatase and the light-dependent enzyme protochlorophyllide oxidoreductase (POR) in angiosperms, including tomato [37,38]. The balance between chlorophyll-a and chlorophyll-b is dynamically controlled by chlorophyllide oxygenase (CAO), whereas the PAO/phyllobilin pathway mediates chlorophyll degradation during senescence. Disruption of pigment biosynthesis or catabolic processes alters chlorophyll homeostasis, resulting in chlorosis or pale-green phenotypes [39].

Carotenoids, including carotenes and xanthophylls, are synthesized through the plastidial methylerythritol phosphate (MEP) pathway and contribute to yellow-orange coloration and play essential photoprotective roles [40]. Phytoene synthase (PSY) and lycopene β-cyclase are key regulators of carotenoid biosynthesis, whereas xanthophyll cycle enzymes, including violaxanthin de-epoxidase (VDE) and zeaxanthin epoxidase (ZEP), enable rapid dissipation of excess excitation energy through non-photochemical quenching [41]. Unlike tomato fruit, where carotenoids determine the characteristic red, orange, or yellow coloration, carotenoids in tomato leaves primarily function in photoprotection, stabilization of photosynthetic complexes, and prevention of photooxidative damage rather than functioning as the principal determinants of leaf color [22]. Anthocyanins are flavonoid-derived pigments that accumulate in vacuoles and confer red to purple pigmentation in leaves in response to developmental and environmental factors. Their biosynthesis is regulated by the MBW transcriptional complex (MYB-bHLH-WD40), which integrates signals from light, temperature, and nutrient availability [42].

**Figure 1:**
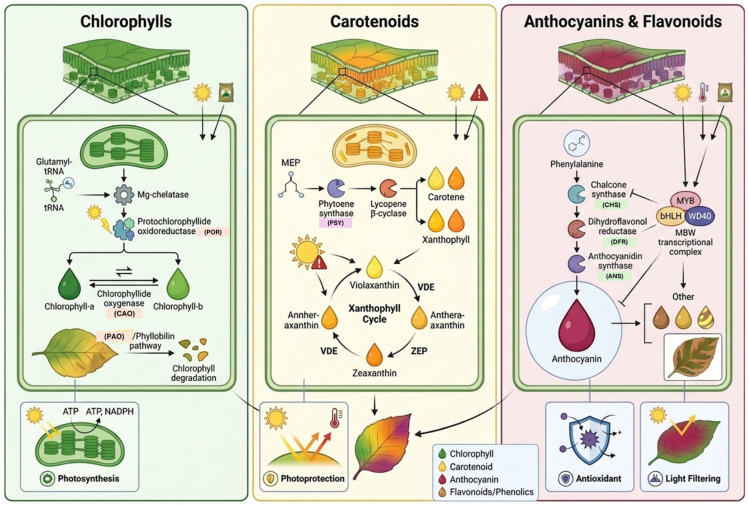
Schematic overview of the biosynthesis, degradation, and physiological functions of the major plant pigment classes, including chlorophylls, carotenoids, and anthocyanins/flavonoids. Chlorophyll biosynthesis is regulated by protochlorophyllide oxidoreductase (POR) and chlorophyllide oxygenase (CAO), whereas chlorophyll degradation during leaf senescence proceeds through the pheophorbide a oxygenase (PAO)/phyllobilin pathway. Carotenoid biosynthesis is initiated by phytoene synthase (PSY), while violaxanthin de-epoxidase (VDE) and zeaxanthin epoxidase (ZEP) regulate the xanthophyll cycle to dissipate excess light energy and protect the photosynthetic apparatus. Anthocyanin and flavonoid biosynthesis originates from the phenylpropanoid pathway through chalcone synthase (CHS), with dihydroflavonol reductase (DFR) and anthocyanin synthase (ANS) catalyzing anthocyanin formation. Collectively, these pigments contribute to photosynthesis, photoprotection, antioxidant defense, light filtering, and plant adaptation to environmental stresses. Figure created using FigureLabs (https://www.figurelabs.ai/) and Microsoft PowerPoint.

Key enzymes such as chalcone synthase (CHS), dihydroflavonol reductase (DFR), and anthocyanidin synthase (ANS) regulate metabolic flux, whereas anthocyanins function as potent antioxidants and light filters under stress conditions, including high irradiance and low temperature [43]. In contrast to several ornamental and cereal species, anthocyanins are not a major determinant of leaf coloration in tomato. Instead, anthocyanin accumulation in tomato leaves is generally restricted to high-anthocyanin genotypes carrying loci such as Aft or is induced by environmental factors, including high light and low temperature [44]. Flavonoids and related phenolic compounds contribute to fine-scale variation in leaf coloration, including bronze or variegated patterns. These metabolites originate from branch points in the phenylpropanoid pathway and are regulated through interactions between developmental regulators and environmental signaling networks [45,46] (Figure 1). The integrated relationships among major pigments, their molecular regulation, physiological functions, and agronomic significance are summarized in Table 1 [45,46].

## 3. Color Phenotypes of Tomato Leaves: Physiological and Molecular Mechanisms

Leaf color is recognized as a functional trait when it reflects physiological processes associated with photosynthetic performance, resource-use efficiency, stress tolerance, and plant productivity [59]. However, leaf color alone is not a direct indicator of plant performance, as similar color phenotypes can result from different genetic and environmental factors [60]. Therefore, leaf color should be considered a functional trait only when supported by evidence demonstrating that pigment composition or chloroplast characteristics are associated with measurable physiological or ecological performance [61]. Tomato leaves show diverse color phenotypes with distinct pigment and physiological features, as discussed in the following sections.

### 3.1. Dark Green Leaves

Dark green phenotypes in tomato are generally associated with increased chlorophyll accumulation and alterations in chloroplast biogenesis and thylakoid membrane organization [62]. Studies in tomato have shown that mutations affecting plastid regulatory pathways and chloroplast biogenesis can produce a significant dark green phenotype characterized by increased chlorophyll content and altered chloroplast ultrastructure. For example, the tomato high pigment (hp) mutants, including *hp1* and *hp2*, exhibit increased chloroplast content and enhanced chlorophyll accumulation in leaves and immature fruits [47,48]. These phenotypes result from altered light signal transduction associated with mutations in UV-Damaged DNA Binding Protein 1 (*DDB1*) and de-etiolated1 (*DET1*), which regulate plastid development and photomorphogenesis [63]. Loss of function mutations in *DDB1* and *DET1* enhance light-responsive gene expression, stimulate chloroplast proliferation, and promote the transcription of photosynthesis-associated genes (*PhANGs*), thereby increasing chlorophyll accumulation and leaf greenness [49]. Similarly, studies of tomato chloroplast regulatory mutants have shown that increased chlorophyll content is frequently accompanied by enhanced thylakoid membrane development and alterations in grana organization [64]. Mechanistically, increased chlorophyll accumulation may result from coordinated regulation of tetrapyrrole biosynthesis and the chlorophyll turnover pathway. In contrast, the assembly and maintenance of light-harvesting chlorophyll a/b-binding (LHC) proteins promote efficient energy capture [65]. Altered expressions of LHC proteins and other photosystem-associated components can modify antenna size and excitation energy distribution, thereby influencing photosynthetic performance and leaf coloration [66]. Dark green leaves often have higher chlorophyll content; however, evidence from crop canopy studies indicates that significant reductions in chlorophyll can occur with little effect on canopy photosynthesis [67]. Photosynthetic performance depends on multiple factors, including mesophyll conductance, chloroplast function, canopy structure, and source-sink balance [68]. In addition, nitrogen availability indirectly modulates chlorophyll accumulation and leaf greenness because a significant proportion of leaf nitrogen is invested in photosynthetic machinery, including Rubisco and chlorophyll-protein complexes in the thylakoid membrane [69]. Consequently, nitrogen status can influence protein accumulation and leaf greenness; however, this relationship is primarily regulatory and resource-driven rather than causally directing chloroplast structural expansion [70].

### 3.2. Light Green Leaves

Light and pale green leaf phenotypes are associated with reduced chlorophyll accumulation resulting from disruptions in chlorophyll metabolism and/or impaired chloroplast development [71]. At the metabolic level, decreased chlorophyll content may result from reduced activity of enzymes involved in the tetrapyrrole biosynthetic pathway. These enzymes include glutamyl-tRNA reductase (GluTR), which catalyzes the first committed step of the C5 pathway leading to 5-aminolevulinic acid (ALA) biosynthesis, and magnesium chelatase, which catalyzes the insertion of Mg into protoporphyrin IX, committing the tetrapyrrole pathway to chlorophyll biosynthesis [37,72]. Mutations or reduced expressions of genes encoding these enzymes have been shown to decrease chlorophyll accumulation and generate chlorotic or pale-green phenotypes in various plant species [73]. The tomato ghost (gh) mutant, caused by defects in plastid terminal oxidase (PTOX), disrupts carotenoid biosynthesis and plastoquinone redox homeostasis, resulting in secondary defects in chloroplast development and pigment accumulation. These alterations lead to pale-green tissues and reduced photosynthetic efficiency [51]. Recent studies have shown that GENOMES UNCOUPLED 4 (*GUN4)*, SLOW GREEN 1 (*SG1)*, Mg-chelatase H subunit (*CHLH)*, and Tetratricopeptide Repeat 4 (*TPR4)* genes from a chloroplast-associated regulatory complex promote chlorophyll biosynthesis and pigment accumulation, thereby facilitating chloroplast development and maintaining leaf greenness [52]. Nuclear-encoded genes regulating chloroplast biogenesis are essential for proper chloroplast differentiation and leaf greenness. For example, mutations in genes encoding the TOC/TIC protein import complexes, such as *TOC33* and *TIC40*, impair the import of nucleus-encoded proteins in chloroplasts, resulting in defective chloroplast development [53]. Similarly, mutation in the *FtsH* proteases disrupts thylakoid membrane assembly and photosystem stability [54]. At the physiological level, reduced chlorophyll concentration decreases light-absorbing capacity and can alter the chlorophyll-to-carotenoid ratio, resulting in a lighter green phenotype [74]. Because chlorophyll accumulation is tightly coordinated with photosystem assembly and thylakoid development, decreased pigment content is frequently accompanied by reduced photosynthetic performance. However, the extent of functional limitation depends on the severity of the developmental defect and environmental conditions [75].

### 3.3. Yellow and Chlorotic Leaves

Yellowing and chlorosis generally occur when chlorophyll degradation exceeds chlorophyll biosynthesis, resulting in a net loss of photosynthetic pigments [76]. At the mechanistic level, chlorophyll catabolism proceeds through the conserved PAO/phyllobilin pathway, in which chlorophyll is sequentially degraded after its release from photosynthetic complexes [77]. Chlorophyll degradation during leaf senescence is mainly regulated by the conserved pheophorbide a oxygenase/phyllobilin (PAO/phyllobilin) pathway, resulting in the formation of non-phototoxic phyllobilins [78].This process involves STAY-GREEN (SGR)-mediated Mg de-chelation, pheophytinase (PPH)-catalyzed dephytylation, and PAO-dependent cleavage of the tetrapyrrole ring. In this pathway, stay-green (SGR)/non-yellowing 1 (NYE1) functions as a key regulatory factor by promoting disassembly of the light-harvesting complex II (LHCII) and coordinating chlorophyll catabolic enzymes, including non-yellow coloring 1 (NYC1), 7-hydroxymethyl chlorophyll a reductase (HCAR), PPH, PAO, and red chlorophyll catabolite reductase (RCCR) [79,80]. In Arabidopsis, *SGR1* acts upstream of these enzymes to ensure efficient chlorophyll breakdown, a mechanism also conserved in crops such as rice and tomato [81]. Chlorosis is further intensified under nutrient limitation through the disruption of chlorophyll metabolism. Nitrogen deficiency constrains aminolevulinic acid synthesis and chlorophyll-protein complex formation, whereas magnesium deficiency impairs Mg-chelatase activity and limits Mg incorporation into protoporphyrin IX, thereby restricting chlorophyll biosynthesis and chloroplast stability [82,83]. Environmental stress also promotes the accumulation of ROS and activates hormone-mediated signaling pathways involving abscisic acid (ABA) and ethylene, which in turn induce senescence-associated transcriptional programs and chlorophyll catabolic genes, accelerating photosystem disassembly and pigment loss [84]. For example, drought- and salinity-induced leaf yellowing are frequently associated with increased expression of *SGR*, *NYC1*, *PPH*, and *PAO*, whereas nitrogen deficiency commonly induces progressive chlorosis through coordinated suppression of chlorophyll biosynthesis and enhanced nutrient remobilization during leaf senescence [85]. Chlorosis is a nonspecific visual symptom that results from nutrient deficiencies, pathogen infection, salinity, drought, senescence, herbicide injury, or genetic defects affecting chloroplast development [86]. Therefore, advanced diagnostic approaches are required, supported by physiological, biochemical, or molecular evidence, to determine the underlying cause.

### 3.4. Purple and Reddish Leaves

Purple and reddish leaf phenotypes are physiological responses to enhanced anthocyanin accumulation within vacuoles, particularly in epidermal and subepidermal tissues [87]. This pigmentation pattern has been documented across diverse plant taxa, including *Arabidopsis thaliana*, *Zea mays*, *Oryza sativa*, and various ornamental and woody species, where it is commonly induced by abiotic stresses such as low temperature, high light intensity, and phosphorus (P) deficiency [88,89]. At the mechanistic level, these phenotypes are primarily regulated through transcriptional reprogramming of the phenylpropanoid and flavonoid biosynthetic pathways [90]. Stress perception triggers regulatory networks involving MYB-bHLH-WD40 transcription factor complexes, which coordinately upregulate key structural genes, including chalcone synthase (*CHS*), chalcone isomerase (*CHI*), flavanone 3-hydroxylase (*F3H*), and dihydroflavonol 4-reductase (*DFR*), leading to enhanced anthocyanin biosynthesis [9]. Anthocyanin biosynthesis in plants, including tomato, is mainly controlled at the transcriptional level by R2R3-type MYB (R2R3-MYB) transcription factors such as Anthocyanin 2-like (SlAN2-like; Anthocyanin fruit (Aft) locus) and Anthocyanin 2 (SlAN2). These regulators activate late biosynthetic genes, including *dihydroflavonol 4-reductase* (*DFR*), *anthocyanidin synthase* (*ANS*), and *UDP-glucose*: *flavonoid 3-O-glucosyltransferase* (*UFGT*), to promote pigment accumulation in tissues such as fruit peel and vegetative organs under specific conditions [91]. In contrast, MYB repressors such as *SlMYBATV* and *SlMYBL2* negatively regulate the pathway by suppressing transcription of anthocyanin biosynthetic genes, resulting in reduced pigment accumulation [92]. In phosphorus-deficient plants, increased anthocyanin accumulation has been consistently reported in maize and Arabidopsis, often associated with reduced growth rates and altered carbon allocation patterns, suggesting a trade-off between stress defense and biomass accumulation [93,94,95]. Functionally, anthocyanins localized in vacuoles and epidermal tissues act as effective optical filters, attenuating excessive incident radiation and thereby reducing chloroplast photoinhibition under high irradiance or chilling conditions [96]. This screening effect has been demonstrated in red-leaf phenotypes of several crop and model species, where anthocyanin-rich tissues exhibit reduced chlorophyll excitation pressure and improved maintenance of photosystem II (PSII) efficiency under stress [97]. In addition, anthocyanins contribute to cellular redox homeostasis by directly scavenging ROS, including superoxide radicals and hydrogen peroxide, and by modulating antioxidant enzyme activities such as superoxide dismutase and catalase [98]. Collectively, these functions position anthocyanin accumulation as a photoprotective and antioxidative strategy that enhances plant resilience under environmental constraints [99]. However, anthocyanin accumulation does not always indicate enhanced stress tolerance, as it may also reflect stress-induced metabolic reprogramming [100].

### 3.5. White and Albino Leaves

White and albino phenotypes in tomato represent extreme disruptions in chloroplast development, typically arising from defects in plastid biogenesis, gene expression within plastids, or the impact of nuclear-encoded chloroplast proteins [101]. These phenotypes are characterized by a severe loss of photosynthetic capacity, reflecting failures at multiple stages of chloroplast differentiation [102]. In several cases, mutations affecting plastid-encoded RNA polymerase (PEP) subunits or sigma factors (SlG2) severely compromise plastid transcription, leading to reduced expression of photosynthesis-related genes [103,104]. Defects in chloroplast ribosome biogenesis or translation factors, including pentatricopeptide repeat (PPR) proteins required for RNA processing, also frequently result in albino or seedling-lethal phenotypes. As a consequence of these disruptions, chlorophyll biosynthesis is drastically reduced because key enzymes and structural components of the photosynthetic apparatus fail to accumulate or assemble properly [105]. Carotenoid production is often simultaneously impaired due to the absence of functional plastid membranes, further contributing to the loss of green pigmentation [106]. In tomato mutants such as pale-green or early lethal albino lines, seedlings emerge white or translucent and typically fail to sustain autotrophic growth beyond early developmental stages [101]. Tomato white virescent mutants (WV) exhibit pale or white young leaves with reduced chlorophyll content and poorly developed chloroplast ultrastructure, including underdeveloped thylakoid membranes, reflecting defective chloroplast differentiation during early leaf development [32]. Similar albino phenotypes have been reported in mutants affecting chloroplast ribosome function or plastid gene expression, such as albino seedling lethality, where defects in the chloroplast 30S ribosomal protein S1 impair chloroplast ribosome biogenesis and block early chloroplast development [107].

### 3.6. Variegated Leaves

Variegated leaf phenotypes result from spatial heterogeneity in chloroplast development and function, producing distinct sectors with contrasting pigmentation within the same leaf. These patterns commonly result from genetic chimerism, defects in plastid biogenesis, instability in plastid genome inheritance, or mutations in nuclear and plastid-encoded genes involved in chloroplast development, maintenance, and differentiation [108,109]. Green sectors generally contain fully differentiated and photosynthetically competent chloroplasts, whereas white and yellow sectors contain plastids that are arrested at different stages of development and therefore fail to synthesize chlorophyll [110]. At the molecular level, variegation frequently results from the disruption of nuclear genes that regulate chloroplast biogenesis and maintenance. These genes encode proteins involved in plastid transcription, translation, protein import, thylakoid membrane assembly, redox homeostasis, and proteostasis [111]. A widely accepted mechanistic framework for variegation in the threshold model of chloroplast biogenesis, which proposes that developing plastids must surpass a functional threshold to differentiate into photosynthetically active chloroplasts [112]. Plastids that successfully maintain redox homeostasis and photosynthetic complex assembly differentiate into chloroplasts and contribute to green sectors, whereas plastids that fail to reach this developmental threshold remain defective and generate non-green tissue [34]. Evidence from the Arabidopsis variegation mutants *immatuns* (*im*), *spotty*, *var1*, and *var2* supports the model that photosynthetic redox imbalance controls leaf sectoring, and early disruptions in chloroplast redox homeostasis and thylakoid membrane formation can trigger the formation of stable green and white leaf sectors [34]. Plastid genome segregation and developmental lineage also contribute to sector establishment. During leaf expansion, plastids repeatedly replicate and partition among daughter cells. Unequal inheritance of functional and defective plastids, including heteroplasmic plastid populations, can generate clonal sectors that persist throughout development [113]. Histological analyses of variegated mutants further indicate that white sectors frequently result from arrested differentiation of proplastids into mature chloroplasts rather than plastid loss alone. Retrograde signaling between plastids and the nucleus provides an additional molecular layer regulating variegation. Chloroplasts transmit information on their developmental and physiological status to the nucleus, thereby modulating the expression of photosynthesis-associated nuclear genes and developmental programs [10,114]. In Arabidopsis, mutations in VAR1 (*FtsH5*) and VAR2 (*FtsH2*) cause green and white leaf variegation due to impaired chloroplast development in sectors with reduced FtsH activity, leading to defective thylakoid formation and loss of photosynthetic competence [58]. FtsH-dependent protein quality control systems are conserved in higher plants, including tomato, where chloroplast protease activity is important for maintaining chloroplast stability and photosynthetic efficiency, and defects can result in chlorotic or pale leaf phenotypes under stress conditions [115]. In addition, plastid-to-nucleus retrograde signaling mediated by GENOME UNCOUPLED (GUN) components (gun1-gun5) coordinates nuclear expression of photosynthesis-associated genes with chloroplast development status [116]. Multiple retrograde signaling pathways have been identified, including signals derived from tetrapyrrole metabolism, plastid gene expression, ROS, photosynthetic electron transport, and plastid metabolic intermediates. Impaired plastid function, therefore, modifies local nuclear transcriptional responses and reinforces differential chloroplast development across adjacent tissues [117].

## 4. Environmental Regulation of Leaf Color

Leaf color is a dynamic phenotypic trait that reflects changes in pigment composition, chloroplast development, and physiological status in response to environmental conditions. The relative abundance and distribution of chlorophylls, carotenoids, and anthocyanins primarily determine the visible coloration of leaves [118]. These pigments are tightly regulated through interconnected pathways involving chloroplast development, photosynthetic efficiency, and stress-induced metabolic reprogramming [119]. The accumulation and degradation of these pigments are regulated through complex interactions between genetic mechanisms and environmental factors [120]. Environmental stress frequently alters leaf coloration primarily by converging on a common physiological framework involving impaired chloroplast integrity, reduced photosynthetic performance, oxidative stress, and activation of senescence-related pathways, ultimately leading to visible pigment changes. Consequently, changes in leaf color are widely recognized as indicators of plant acclimation and stress responses [2]. In tomato, leaf color is considered an important indicator of plant physiological status and environmental adaptation. Previous studies have shown that abiotic stresses, including temperature extremes, drought, salinity, excessive light, and nutrient deficiency, influence leaf coloration by altering chlorophyll metabolism, chloroplast development, and photosynthetic performance. These changes commonly result in chlorosis, leaf yellowing, or reduced greenness [121,122].

### 4.1. Light Quantity and Spectral Quality

Light is one of the most important environmental factors regulating leaf coloration because it functions both as an energy source for photosynthesis and as a developmental signal that controls pigment biosynthesis [123]. Variations in light intensity influence chlorophyll accumulation, chloroplast development, and the synthesis of photosynthetic pigments [124,125]. Under low-light conditions, plants often increase chlorophyll content and modify pigment composition to enhance light capture efficiency, resulting in darker green leaves. In contrast, excessive light exposure can induce photoinhibition and ROS accumulation, resulting in chlorophyll degradation and visible leaf yellowing or bleaching [126]. Light quality also plays an important role in regulating leaf coloration. Red and blue wavelengths modulate pigment biosynthesis through photoreceptor-mediated signaling pathways involving phytochromes and cryptochromes, which interact with phytohormonal networks [127]. Blue light has frequently been associated with enhanced anthocyanin accumulation and improved chloroplast development, whereas altered red-to-far-red light ratios in shaded environments may suppress anthocyanin synthesis and modify chlorophyll content. Anthocyanin accumulation under high light conditions is considered an adaptive response contributing to ROS scavenging and excess energy dissipation rather than direct pigment replacement [128]. Studies have shown that in tomato, low light reduces photosynthetic capacity, while excessive light decreases chlorophyll content and alters leaf greenness [129,130].

### 4.2. Temperature Stress

Temperature stress significantly influences leaf color by affecting pigment biosynthesis, chloroplast structure, membrane integrity, and cellular metabolism. Both high and low temperatures alter photosynthetic performance and induce physiological adjustments, often resulting in visible changes in pigmentation [131,132].

#### 4.2.1. Heat Stress

Heat stress commonly causes leaf discoloration by disrupting chlorophyll metabolism and damaging photosynthetic structures [133]. Elevated temperatures may reduce chlorophyll biosynthesis, accelerate chlorophyll degradation, and increase ROS production, collectively impairing chloroplast function and decreasing photosynthetic efficiency [134]. Heat-induced injury to thylakoid membranes can further contribute to chlorosis and premature leaf senescence [135]. In tomato, high temperature stress reduces chlorophyll content, damages chloroplast ultrastructure, and suppresses photosynthetic activity, leading to visible leaf yellowing [136,137]. In some plant species, such as members of the Oleaceae and Poaceae families, carotenoid accumulation increases under heat stress conditions as part of a protective response, as carotenoids dissipate excess energy and mitigate oxidative stress. However, the magnitude and direction of pigment changes under heat stress vary among species and depend on stress duration and intensity [138,139].

#### 4.2.2. Cold Stress

Cold stress frequently induces visible changes in leaf coloration due to alterations in chlorophyll metabolism and the accumulation of secondary pigments [140]. Reduced temperatures often limit chlorophyll synthesis and photosynthetic activity while increasing oxidative stress within leaf tissues [141]. In many species, cold exposure is associated with the accumulation of soluble sugars and the induction of anthocyanin biosynthesis, leading to red, orange, or purple coloration. Anthocyanins are considered to play a photoprotective role under cold conditions by reducing excess light absorption and limiting photo-oxidative damage when photo-synthetic capacity is reduced. Prolonged exposure to low temperatures may also impair chloroplast development and contribute to chlorosis and tissue injury [142,143]. Cold stress induces oxidative stress and triggers anthocyanin accumulation in some cultivars of tomato as an adaptive photosynthetic response, although the extent of pigmentation change depends on genotype and stress severity [144]. For example, the wild tomato *S. habrochaites* LA1777 exhibit stronger stress tolerance, including under cold conditions, compared with the cultivated tomato *S. lycopersicum* (Ailsa Craig), which shows weaker adaptive responses and lower stress resilience [145].

### 4.3. Water-Related Stresses

Water availability significantly influences leaf color because plant water status directly affects photosynthesis, nutrient transport, pigment stability, and oxidative metabolism [146].

#### 4.3.1. Drought

Drought stress commonly alters leaf pigmentation through reductions in chlorophyll content and changes in antioxidant metabolism [147]. Water limitation restricts photosynthetic carbon assimilation and promotes oxidative stress, which can accelerate chlorophyll degradation and impair chloroplast function. As a result, leaves may exhibit chlorosis, yellowing, or progressive discoloration [148]. In tomato, drought stress reduces chlorophyll content and photosynthetic efficiency, leading to leaf wilting and chlorosis due to oxidative damage and impaired chloroplast function [149]. In many plant species, such as tobacco, drought conditions are also associated with increased anthocyanin accumulation, which is considered an adaptive response that enhances ROS scavenging and photoprotection under water deficit conditions [150]. Current evidence suggests that anthocyanins contribute primarily through antioxidant and photoprotective functions, helping reduce oxidative damage and maintain cellular homeostasis during water deficit. Under prolonged or severe drought, extensive chlorophyll loss may ultimately lead to premature senescence [140,151].

#### 4.3.2. Flooding

Flooding and waterlogging create oxygen-deficient conditions around the root system, disrupting water uptake, nutrient acquisition, and energy metabolism [152]. Such hypoxic conditions primarily influence leaf coloration indirectly by impairing mitochondrial respiration and enhancing systemic oxidative stress [153]. These physiological disturbances often impair chloroplast function and reduce chlorophyll accumulation, resulting in pale green or chlorotic leaves. Flood-induced oxidative stress may further alter pigment composition depending on species-specific tolerance mechanisms [154]. Although pigment responses vary among plant species and environmental conditions, prolonged flooding has been shown to reduce photosynthetic performance and alter leaf coloration in white oak species, with the magnitude of these effects depending on species-specific flooding tolerance [155]. In tomato, flooding-induced ethylene (ET) impairs both light and dark reactions of photosynthesis by accelerating the dismantling of the photosynthetic apparatus, leading to chloroplast damage and early senescence [156].

### 4.4. Nutrient Deficiencies

Mineral nutrients are essential for pigment synthesis, chloroplast development, and photosynthetic processes; therefore, nutrient deficiencies often result in characteristic changes in leaf coloration [82]. Unlike environmental stresses that cause transient physiological disruption, nutrient deficiencies regulate leaf color through sustained metabolic reprogramming affecting chlorophyll turnover, photosynthetic protein assembly, and carbon-nitrogen balance [70]. Nitrogen deficiency is one of the most common causes of leaf yellowing because nitrogen is an essential component of chlorophyll molecules and photosynthetic proteins. Reduced nitrogen availability limits chlorophyll biosynthesis and decreases photosynthetic capacity, leading to generalized chlorosis. As nitrogen is mobile within plants, visual symptoms commonly appear first in older leaves as nutrients are redistributed toward younger tissues [85]. Phosphorus deficiency frequently alters leaf color by affecting energy metabolism and carbohydrate transport. Deficient plants may develop dark green, reddish, or purple coloration, particularly in older leaves. This response is often associated with carbohydrate accumulation and the induction of anthocyanin biosynthesis [157]. Potassium deficiency affects numerous physiological processes, including stomatal regulation, enzyme activation, and carbohydrate transport. Deficiency symptoms often include marginal chlorosis and necrosis that begin in older leaves. Although potassium is not a structural component of chlorophyll, insufficient potassium can indirectly induce chlorophyll instability and increase susceptibility to oxidative damage [158]. Magnesium plays a central role in photosynthesis because it forms the central atom of the chlorophyll molecule. Magnesium deficiency commonly results in interveinal chlorosis while leaf veins remain relatively green. Reduced magnesium availability disrupts chlorophyll synthesis and impairs energy transfer processes within chloroplasts, leading to decreased photosynthetic performance [159]. Iron participates in electron transport, and enzymatic reactions are essential for photosynthesis. Because iron mobility within plants is limited, deficiency symptoms typically appear in younger leaves and are characterized by interveinal chlorosis that may progress to severe bleaching under prolonged deficiency [82].

### 4.5. Salinity Stress

Salinity stress alters leaf coloration through the combined effects of osmotic stress, ionic toxicity, nutrient imbalance, and oxidative damage. High salt concentrations often reduce chlorophyll accumulation and impair photosynthesis, leading to reduced leaf greenness and chlorosis [160]. Salinity stress has also been reported to induce anthocyanin accumulation in certain species, such as *Vitis vinifera*, as part of the antioxidant defense system. These pigments can help maintain redox homeostasis and mitigate oxidative damage [161]. In tomato plants, NaCl impacts dynamic stomatal and photosynthetic kinetics by osmotic effects and reduces photosynthetic capacity by ionic effects [162,163]. The extent of pigment alteration under salinity stress depends on species-specific tolerance, developmental stages, environmental conditions, and the ionic composition of the salt stress [164].

### 4.6. Heavy Metal Stress

Heavy metal exposure can significantly alter leaf color through its effect on chloroplast structure, photosynthetic metabolism, and oxidative homeostasis [165]. Metals such as cadmium, lead, mercury, and excess copper interfere with enzymatic pathways involved in chlorophyll biosynthesis and increase ROS production. As a result, affected plants often exhibit chlorosis, reduced leaf greenness, bronzing, and necrotic lesions [166,167]. Studies have shown that in tomato, heavy metals, such as cadmium, damage chloroplast structure, photosynthetic pigments, and chlorophyll-protein complexes, resulting in an overall decrease in efficiency of the carbon assimilation pathway [168,169]. In some species, including tomato, heavy metal stress is associated with anthocyanin accumulation, which contributes to antioxidant protection and stress acclimation. However, prolonged exposure generally leads to progressive pigment loss and reduced photosynthetic function [170,171].

## 5. Hormonal Control of Tomato Leaf Color

**Figure 2:**
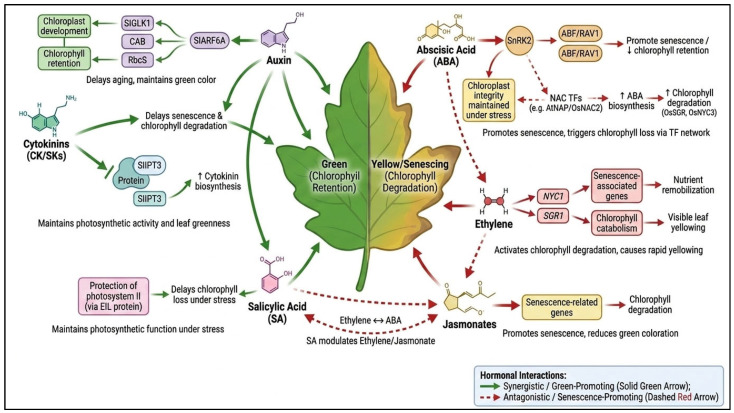
Schematic illustration of hormonal regulation during leaf senescence and leaf color dynamics. The diagram shows the antagonistic interplay between senescence-delaying (green promoting) and senescence-promoting hormonal pathways that regulate chloroplast function and leaf coloration. CKs, auxins, and SA synergistically promote chloroplast development, photosynthetic activity, and chlorophyll stability through regulatory factors such as SlARF6A and SlIPT3, thereby maintaining leaf greenness and delaying senescence. In contrast, ABA, ET, and JA synergistically activate senescence-associated transcription factors and chlorophyll catabolic genes, including *SGR1* and *NYC1*, leading to chlorophyll degradation, nutrient remobilization, and leaf yellowing. The antagonistic crosstalk between these hormones ultimately determines leaf longevity and the progression of senescence. Note: In this review, chlorosis refers to the physiological loss of chlorophyll, leaf yellowing to the visible yellow phenotype resulting from chlorosis or senescence, and leaf bleaching to severe or complete pigment loss producing white or nearly white tissues. Figure created using FigureLabs (https://www.figurelabs.ai/) and Microsoft PowerPoint.

ABA influences leaf appearance mainly by promoting leaf senescence through *SnRK2*-mediated activation of *ABF* and *RAV1* transcription factors, which affects chlorophyll retention rather than directly regulating pigment biosynthesis [172]. ABA signaling regulates photosynthetic activity and chloroplast integrity by modulating chlorophyll degradation under stress conditions. However, its effect on leaf color is mainly indirect through regulation of senescence and chlorophyll catabolic pathways rather than direct control of pigment biosynthesis, consistent with stay-green phenotypes caused by impaired chlorophyll degradation [173]. Changes in ABA status also influence leaf senescence, which ultimately determines chlorophyll retention and leaf greenness [174]. Studies show that ABA effects are context-dependent, as it can promote senescence and chlorophyll degradation under certain conditions [175], while also contributing to stress acclimation and chloroplast protection depending on developmental stage and stress severity [176]. Current evidence does not support a direct role of ABA in regulating pigment biosynthesis in tomato leaves; however, NAC transcription factors of other plant species, such as *AtNAP* and *OsNAC2*, promote leaf senescence by inducing ABA biosynthetic genes, thereby increasing ABA levels that enhance chlorophyll degradation and leaf yellowing [174,177]. *OsNAC2* further regulates chlorophyll degradation genes, including *OsSGR* and *OsNYC3*, indicating that ABA-related pathways influence leaf color mainly through an NAC TF-mediated senescence regulatory network rather than direct control of pigment biosynthesis [178]. ET regulates leaf color change through its well-documented role in promoting leaf senescence [179,180]. It activates chlorophyll catabolic genes such as *NYC1* and *SGR1*, promoting rapid chloroplast degradation, chlorophyll loss, and consequent visible yellowing [181]. Tomato mutants such as Never-ripe (Nr), which is defective in ET perception, exhibit delayed senescence and prolonged maintenance of chlorophyll compared with wild-type plants, demonstrating reduced leaf yellowing due to impaired ET signaling. Conversely, ET overproduction lines in tomato display precocious senescence and earlier chlorophyll degradation [182]. Transcriptomic analyses further show that ET induces senescence-associated genes involved in chlorophyll catabolism and nutrient remobilization, while suppressing photosynthetic gene expression during leaf aging. These ethylene-responsive processes are tightly associated with reduced chlorophyll retention and progressive loss of green coloration [183,184]. Studies show that cytokinins (CKs) help maintain green leaf color in tomato by delaying leaf senescence and reducing chlorophyll degradation. CKs signaling, including root-shoot communication, supports longer photosynthetic activity and preserves chlorophyll in leaves [185]. In tomato, CKs responses indicate that increased cytokinin accumulation, particularly through SlIPT3-mediated cytokinin biosynthesis, supports the maintenance of leaf physiological activity and enhances tolerance to stress conditions. Current evidence suggests that CK-mediated retention of green color is primarily associated with delayed chlorophyll degradation and prolonged leaf function rather than stimulation of new pigment formation [186] (Figure 2).

Studies in tomato suggest that auxins influence leaf color through the regulation of chlorophyll accumulation and photosynthetic activity. The auxin response factor Auxin Response Factor 6A (SlARF6A) promotes chlorophyll retention by activating genes such as Golden2-like 1 (*SlGLK1*), chlorophyll a/b-binding protein (*CAB*), and ribulose-1,5-bisphosphate carboxylase/oxygenase small subunit (*RbcS*), which support chloroplast development and delay leaf aging [50]. Thus, current evidence supports a role for auxins as indirect regulators of tomato leaf color through developmental control of leaf longevity and photosynthetic maintenance [187]. JA signaling has been associated with the activation of senescence-related responses and a decline in photosynthetic performance, resulting in the visible loss of green coloration during leaf aging. Studies suggest that jasmonates contribute to chlorophyll degradation during senescence rather than acting as direct regulators of pigment biosynthesis [188,189]. Salicylic acid (SA) is primarily reported as a stress- and senescence-regulated signaling molecule that helps maintain photosynthetic function and delays chlorophyll loss. For example, SA-mediated protection of PSII via Ethylene Insensitive 3-like (EIL) protein signaling under chilling stress has been associated with sustained chlorophyll levels. Therefore, SA is considered an indirect regulator of tomato leaf color through modulation of stress responses and senescence processes [190]. Collectively, available studies indicate that interactions among hormonal pathways regulate the timing and progression of leaf senescence and chlorophyll turnover, thereby influencing chlorophyll retention and ultimately determining visible changes in tomato leaf coloration.

## 6. Leaf Color as a Biomarker of Plant Health and Stress

**Figure 3:**
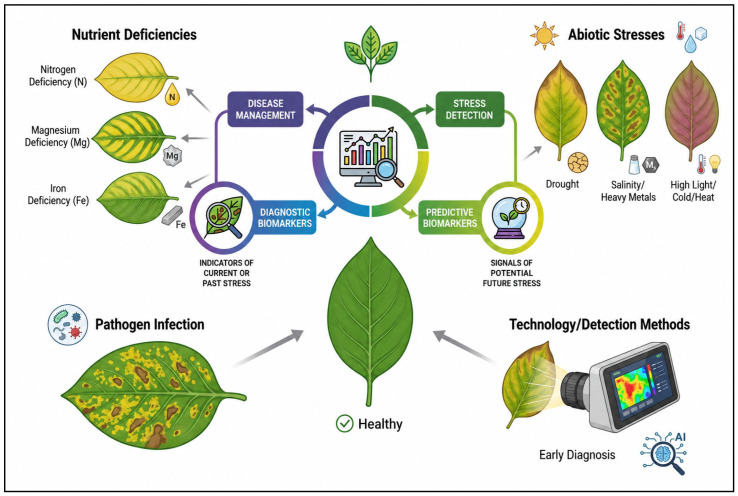
Schematic illustration of the major factors affecting plant health and the application of modern technologies for stress diagnosis and management. Plant stressors are categorized into three major groups: nutrient deficiencies, abiotic stresses, and pathogen infections caused by biological agents such as bacteria and fungi. The central framework highlights the integration of AI-based technologies and biomarker analysis for the early detection and monitoring of plant stress. These approaches facilitate both diagnostic assessment of existing or previous stress conditions and predictive analysis of potential future stress, thereby supporting timely disease management, improved crop protection, and enhanced agricultural sustainability. Figure created using FigureLabs (https://www.figurelabs.ai/) and Microsoft PowerPoint.

Leaf color is one of the earliest and most visible indicators of plant physiological status and is widely used as a biomarker for monitoring plant health. Changes in leaf color largely reflect alterations in photosynthetic pigments and chloroplast function and often occur before a visible reduction in growth and productivity [118,191] (Figure 3). Environmental and biological stresses can modify pigment metabolism and activate pathways associated with chlorophyll biosynthesis, degradation, and stress-responsive signaling [133]. Therefore, quantitative assessment of leaf color provides a practical approach for assessing plant metabolic activity and early stress responses. Leaf color changes are widely used for the early diagnosis of nutrient imbalance because nutrient deficiency often disrupts pigment synthesis and photosynthetic metabolism before severe morphological symptoms appear [192]. Nitrogen deficiency commonly induces generalized chlorosis due to accelerated chlorophyll degradation and remobilization of nitrogen from older leaves to developing tissues. Molecular studies have shown that nitrogen starvation activates senescence-associated pathways and induces the expression of chlorophyll catabolic genes, including *SGR*, *NYC1*, and *PAO*, resulting in chloroplast degradation and leaf yellowing [193]. Magnesium deficiency causes chlorosis because Mg serves as the central atom in chlorophyll molecules. In contrast, iron deficiency impairs chlorophyll biosynthesis by limiting enzymatic reactions in the tetrapyrrole pathway, often resulting in interveinal chlorosis in young leaves [194]. Distinct nutrient deficiencies generate characteristic spatial color signatures that can be associated with nutrient mobility and molecular function. Recent studies integrating leaf color analysis with chlorophyll fluorescence and machine-learning approaches have demonstrated high accuracy in the early detection and discrimination of nutrient disorders before measurable reductions in biomass [195]. Abiotic stresses such as drought, salinity, extreme temperatures, heavy metal toxicity, and excessive light intensity often manifest as changes in leaf color by disrupting chlorophyll metabolism and reducing photosynthetic efficiency [196].

Stress conditions alter both chlorophyll biosynthesis and degradation pathways through the regulation of key enzymes, including glutamyl-tRNA reductase (HEMA), magnesium chelatase (CHLH), protochlorophyllide oxidoreductase (POR), and chlorophyll degradation. In addition to environmental regulation, leaf color variation in tomato is significantly influenced by genetic determinants that regulate chloroplast development, pigment biosynthesis, and senescence processes. For example, *hp1*, *hp2*, *hp3*, and *hp2j* mutants have enhanced plastid biogenesis and pigment accumulation [197]. The *ghost* (PTOX) mutant affects chloroplast redox balance and causes photobleaching phenotypes [51], and *SlGLK1*/*SlGLK2*-mediated regulation of chloroplast development and photosynthetic competence [198]. Alterations in chloroplast function have also been linked to pentatricopeptide repeat (PPR) proteins involved in chloroplast RNA editing [199], as well as mutations in MEP pathway enzymes such as 1-deoxy-D-xylulose-5-phosphate synthase (DXS) that impair isoprenoid-derived pigment biosynthesis [200]. In addition, ethylene-related signaling mutants such as Never-ripe influence senescence-associated chlorophyll degradation and delayed leaf yellowing [201]. Accumulation of carotenoids and anthocyanins may also occur as photoprotective responses, leading to yellow, purple, or reddish leaf phenotypes [202]. Because pigment alterations often precede irreversible physiological damage, leaf color serves as an effective early biomarker for detecting abiotic stress and monitoring plant adaptive responses under adverse environmental conditions [191]. Many bacterial, fungal, and viral pathogens target chloroplast-associated processes to suppress photosynthesis and redirect host metabolism. Infection often results in chlorosis, mosaic patterns, necrosis, and localized yellowing before severe disease symptoms become visible [203,204]. At the molecular level, pathogen-induced activation of stress-related phytohormone signaling pathways, including SA, JA, ethylene, and ABA, directly or indirectly regulates the expression of genes encoding chlorophyll degradation and ROS metabolism enzymes, thereby altering photosynthetic processes and accelerating leaf discoloration [205]. Chloroplast-generated ROS act as both signaling and oxidative molecules under stress conditions, triggering retrograde chloroplast-to-nucleus signaling pathways that coordinate immune and stress responses while also contributing to oxidative damage, chloroplast dysfunction, and pigment degradation [206]. Pathogen-triggered activation of senescence-related genes and chlorophyll degradation pathways generate distinct color signatures that can be detected using optical sensors and imaging technologies. Consequently, quantitative leaf color assessment provides a rapid and non-invasive approach for early disease detection and crop protection management [207,208].

## 7. Future Directions and Research Prospects

Although considerable progress has been made in understanding the physiological and molecular basis of tomato leaf coloration, several important knowledge gaps remain, limiting its translation into breeding and precision agriculture applications. Future research should therefore move beyond descriptive characterization of color phenotypes and adopt predictive, mechanistic, and integrative frameworks that connect leaf pigmentation dynamics with whole-plant performance under fluctuating environmental conditions [209]. Current understanding of tomato leaf color largely relies on independent studies of transcriptomic, physiological, or biochemical responses [210,211]. Future research should emphasize integrated multi-omics approaches, particularly metabolomics combined with genomics, transcriptomics, proteomics, and epigenomics, to reveal regulatory networks and metabolic pathways that regulate leaf pigmentation and improve overall plant performance [212]. Such integration would enable identification of key molecular hubs that coordinate chloroplast development, pigment metabolism, hormone signaling, and environmental adaptation [213]. In tomato, genomic and functional analyses of the *Aft* locus have already identified the R2R3-MYB transcription factor SlAN2-like as a central regulator of anthocyanin biosynthesis through the MBW complex [214]. Transcriptomic studies in high-anthocyanin *Aft* and atroviolacea (*atv*) tomato lines further revealed coordinated upregulation of phenylpropanoid and anthocyanin biosynthetic genes, including *CHS* and *DFR*, resulting in enhanced pigment accumulation [215]. In addition, metabolomic analyses under high light and low temperature stress demonstrated increased flavonoid and anthocyanin levels, linking transcriptional changes with metabolite accumulation [216]. Particular attention should be given to temporal and spatial multi-omics analyses to capture developmental transitions and tissue-specific regulation of leaf color [217,218]. Leaf coloration is not uniformly distributed across tissues and often reflects localized differences in chloroplast differentiation, pigment accumulation, and stress signaling [219]. Emerging approaches such as single-cell RNA sequencing, spatial transcriptomics, and spatial metabolomics provide opportunities to characterize cellular heterogeneity associated with chlorotic, variengated, or anthocyanin-rich sectors [220]. Applying these technologies in tomato could reveal previously unresolved interactions among mesophyll cells, epidermal tissues, vascular tissues, and plastid populations during color establishment. As cited above, although numerous candidate genes associated with leaf color have been identified, their causal relationships remain insufficiently validated. Future studies should combine CRISPR-Cas genome editing, base editing, and inducible gene expression systems to dissect the function of chloroplast regulators, pigment biosynthetic enzymes, retrograde signaling components, and hormone-responsive transcription factors [221,222]. Gene editing strategies targeting chlorophyll turnover, chloroplast maintenance, and photoprotective pathways may also facilitate the development of tomato cultivars with improved photosynthetic efficiency and enhanced environmental resilience [223]. Leaf color is highly responsive to environmental fluctuations; however, the mechanistic understanding of genotype-environment interactions remains limited [224]. Future work should employ controlled environment platforms and field-based phenotyping to quantify how temperature, water availability, salinity, nutrient status, and light conditions reshape pigment dynamics across diverse tomato genotypes. Long-term studies integrating climate variability are particularly important for predicting crop performance under changing environmental conditions [225]. Advances in imaging technologies, particularly when integrated with AI and machine learning, provide opportunities to convert leaf color from a qualitative trait into a quantitative physiological indicator through high-throughput phenotyping and data-driven analysis [226]. Future research should integrate RGB imaging, hyperspectral sensing, chlorophyll fluorescence imaging, thermal imaging, and machine learning algorithms to develop robust predictive models of plant health. Combining imaging data with molecular and physiological measurements may enable early detection of nutrient deficiencies, abiotic stress, and disease before visible yield losses occur [195,227]. Future breeding programs should exploit leaf color traits not merely as visual markers but as indicators of physiological performance and stress adaptation. A major long-term objective should be the development of predictive frameworks that link pigment dynamics with photosynthesis, growth, and yield formation. Coupling physiological modeling with artificial intelligence and digital twin approaches may enable the simulation of leaf color responses under variable environmental conditions. Such predictive systems could support decision-making in breeding, crop management, and climate-resilient agriculture [228].

## 8. Conclusions

Tomato leaf color is a complex trait shaped by pigment metabolism, chloroplast function, genetic and hormonal regulation, and environmental influences. These interactions produce diverse phenotypes that reflect photosynthesis, plant health, and stress adaptation. Recent advances in omics and imaging technologies have improved our understanding of regulatory networks and demonstrated the strong connection between leaf coloration and crop performance, highlighting a more integrated mechanistic framework linking pigment biosynthesis, chloroplast development, and stress-responsive signaling pathways. Leaf color is now recognized as a functional indicator of plant physiological status with strong potential for early stress detection and precision agriculture applications. Despite these advances, several key unresolved questions remain, particularly regarding how molecular-level changes translate into field-scale phenotypes under fluctuating environmental conditions and how multiple stresses interact to shape leaf color expressions. However, gaps remain in linking molecular mechanisms with whole-plant and field-scale responses. From an applied perspective, a deeper understanding of these mechanisms offers important opportunities for tomato breeding, especially for developing cultivars with improved stress tolerance, optimized pigment composition, and enhanced performance in controlled-environment agriculture systems. Integrative approaches combining systems biology, high-throughput phenotyping, and predictive modeling will be essential to translate leaf color research into improved tomato productivity and resilience under changing environmental conditions.

## Figures and Tables

**Table 1:** Tomato leaf color phenotypes: molecular basis, physiological effects, and agronomic relevance.

Pigment/Leaf Color Phenotype	Tomato-Specific Mutants/Genes or Loci	Major Molecular Mechanisms	Whole-Plant Physiological Responses	Agronomic Significance	References
Chlorophyll (Dark green)	*hp1*, *hp2*, *DET1*, *DDB1*, *GLK* homologs	Enhanced chlorophyll biosynthesis; increased chloroplast biogenesis; DET1/DDB1-mediated light signaling; GLK-driven chloroplast development; improved thylakoid organization	Higher photosynthetic efficiency, increased light harvesting, enhanced carbon assimilation, and delayed senescence	Increased biomass, improved canopy photosynthesis, potential yield enhancement; marker of vigorous phenotypes	[47,48,49,50].
Reduced chlorophyll (Light green)	*gh*, *PTOX*, *GUN4*, *CHLH*, *TOC33*, *TIC40*, *FtsH*	Impaired tetrapyrrole biosynthesis; defective plastoquinone redox cycling; disrupted chloroplast protein import and proteostasis; impaired thylakoid assembly	Reduced chloroplast function, lower photosynthetic capacity, and decreased energy production	Reduced vigor and productivity; indicator of chloroplast developmental defects and stress sensitivity	[51,52,53,54].
Chlorophyll degradation (Yellow/Chlorotic)	*SlSGR1*, *SlNYC1*, *SlPPH*, *SlPAO*	Activation of PAO/phyllobilin pathway; SGR-mediated Mg dechelation; PPH-mediated dephytylation; ABA/ethylene and nutrient stress signaling	Accelerated senescence, chloroplast dismantling, reduced photosynthesis, nutrient remobilization	Early indicator of nitrogen/magnesium deficiency, drought, salinity, and disease; associated with yield loss	[55]
Anthocyanins (Purple/Red)	*Aft locus*, *SlAN2-like*, *SlAN2*, *SlMYBATV*	Activation of MYB–bHLH–WD40 complex; induction of CHS, CHI, DFR, ANS, UFGT; stress-responsive transcriptional regulation	ROS scavenging, photoprotection, improved oxidative stress tolerance, reduced photoinhibition	Biomarker of cold, high light, and phosphorus deficiency; potential trait for stress-resilient breeding	[56,57]
Carotenoids (yellow/orange contribution in leaves)	*PSY*, *LCYB*, *VDE*, *ZEP*	MEP pathway-driven carotenoid biosynthesis; xanthophyll cycle regulation; photoprotective pigment accumulation	Energy dissipation, photoprotection, antioxidant defense, stabilization of photosystems	Enhanced tolerance to heat, drought, and high light; supports sustained photosynthesis under stress	[41]
White/Albino leaves	PEP-related genes, PPR proteins, ribosomal genes (e.g., chloroplast 30S defects), TOC/TIC components	Severe disruption of plastid gene expression, protein import, and chloroplast ribosome biogenesis; failure of chloroplast differentiation	Loss of photosynthetic capacity, inability to sustain autotrophic growth, and seedling lethality in severe cases	Strong negative impact on survival and yield; key genetic material for chloroplast biogenesis studies	[31,53]
Variegated leaves	*variegated* (*var*) mutants, *FtsH5/VAR1*, *FtsH2/VAR2*, plastid segregation mutants	Defective chloroplast protein quality control; impaired FtsH protease activity; disrupted retrograde signaling; uneven plastid inheritance	Sectoral differences in photosynthetic capacity, altered redox homeostasis, and localized chloroplast dysfunction	Limited direct agronomic value; important model for plastid-nuclear signaling and chloroplast development	[53,58].

## Data Availability

All data supporting the findings of this study are included within the article.
